# Influence of preoperative nucleus pulposus status and radiculopathy on outcomes in mono-segmental lumbar total disc replacement: results from a nationwide registry

**DOI:** 10.1186/1471-2474-12-275

**Published:** 2011-12-02

**Authors:** Thomas Zweig, Christoph Hemmeler, Emin Aghayev, Markus Melloh, Christian Etter, Christoph Röder

**Affiliations:** 1 Institute for Evaluative Research in Medicine, University of Bern, Stauffacherstrasse 78, 3014 Bern, Switzerland; 2 Department of Orthopedic Surgery, University of Otago, Private Bag 1921, Dunedin, New Zealand; 3 Department of Spine Surgery, Hirslanden Clinic, Schänisweg, 5001 Aarau, Switzerland

## Abstract

**Background:**

Currently, herniated nucleus pulposus (HNP) with radiculopathy and other preconditions are regarded as relative or absolute contraindications for lumbar total disc replacement (TDR). In Switzerland it is left to the surgeon's discretion when to operate. The present study is based on the dataset of SWISSspine, a governmentally mandated health technology assessment registry. We hypothesized that preoperative nucleus pulposus status and presence or absence of radiculopathy has an influence on clinical outcomes in patients treated with mono-segmental lumbar TDR.

**Methods:**

Between March 2005 and April 2009, 416 patients underwent mono-segmental lumbar TDR, which was documented in a prospective observational multicenter mode. The data collection consisted of perioperative and follow-up data (physician based) and clinical outcomes (NASS, EQ-5D).

Patients were divided into four groups according to their preoperative status: 1) group degenerative disc disease ("DDD"): 160 patients without HNP and no radiculopathy, classic precondition for TDR; 2) group "HNP-No radiculopathy": 68 patients with HNP but without radiculopathy; 3) group "Stenosis": 73 patients without HNP but with radiculopathy, and 4) group "HNP-Radiculopathy": 132 patients with HNP and radiculopathy. The groups were compared regarding preoperative patient characteristics and pre- and postoperative VAS and EQ-5D scores using general linear modeling.

**Results:**

Demographics in all four groups were comparable. Regarding the improvement of quality of life (EQ-5D) there were no differences across the four groups. For the two main groups DDD and HNP-Radiculopathy no differences were found in the adjusted postoperative back- and leg pain alleviation levels, in the stenosis group back- and leg pain relief were lower.

**Conclusions:**

Despite higher preoperative leg pain levels, outcomes in lumbar TDR patients with HNP and radiculopathy were similar to outcomes in patients with the classic indication; this because patients with higher preoperative leg pain levels benefit from a relatively greater leg pain alleviation. The group with absence of HNP but presence of radiculopathy showed considerably less benefits from the operation, which is probably related to ongoing degenerative processes of the posterior segmental structures. This observational multicenter study suggests that the diagnoses HNP and radiculopathy, combined or alone, may not have to be considered as absolute or relative contraindications for mono-segmental lumbar TDR anymore, whereas patients without HNP but with radiculopathy seem to be suboptimal candidates for the procedure.

## Background

As long as tissue engineering of the intervertebral disc remains in its infancy [1,2] and interbody fusion in younger patients stays under special scrutiny, there will be ongoing enthusiasm for motion preserving procedures as alternative treatment in degenerative disc disease (DDD). Because of reported long convalescence periods after spinal fusion and a presumed risk of adjacent level degeneration, patients increasingly want to be informed about total disc replacement (TDR) especially in case of a herniated nucleus pulposus (HNP). Looking at the published results for the classic posterior procedures such as conventional discectomy, microdiscectomy and percutaneous measures for treating HNP we observe heterogeneous results. Reported re-operation rates up to 18% (0-18%) depending on follow up interval [3,4] and persistent back or leg pain in 6 to 43% [3] of cases emphasize the necessity for clear-cut indications. However, the most recent reasearch confirms good outcomes in patients meeting specific inclusion criteria [5,6].

The aforementioned facts outline demands for a single staged solution with sustainable long term results. Success rates of only 75 to 80% for the classic posterior procedure (lumbar discectomy) might still be challenged [7].

The Food and Drug Administration (FDA) approved the first TDR for the lumbar spine, the SB Charité/DePuy Spine in October 2004 [8,9] followed by the approval of the Synthes ProDisc-L in August 2006 [10]. Considering publications by TDR pioneers [11-13] and studies leading to FDA approval, indications and contraindications for TDR were defined [8,14,15].

The generally accepted indications are:

Single-level DDD between L4-S1 confirmed by MRI, CT and/or CT myelography

Discogenic low back pain at the segment to be operated concordant with pain during provocative discogram

Back and/or leg pain without neural compression

Age between 18 and 60 years

Unsuccessful conservative therapy of at least six months duration [15]

Contraindications are determined as follows:

Central or lateral recess stenosis

Facet joint arthrosis

HNP with neural compression, spondylolisthesis and spondylolysis

Scoliosis

Osteopenia

There are another about 50 contraindications according to the work of Wong et al. [16].

However, the current literature presents only a few studies dealing with the prevalence of indications and contraindications for TDR. Huang et al. reviewed a single-surgeon series of 100 consecutive lumbar patients including solely 5 TDR cases [17]. Fras and Auerbach presented a retrospective 15-months record review of patients in a private clinic indicating that less than 5% of 190 consecutive spine surgery patients were free of contraindications, qualifying them to be potential candidates for TDR [18]. Applying similar criteria Chin [15] evaluated a population of a university outpatient clinic reporting 5-9% of patients without contraindications.

This suggests the need for randomized controlled trials (RCTs) comparing fusion techniques or posterior techniques with motion preserving procedures, but their implementation remains difficult because of various limitations [10,19-22].

In the history of TDR development many biomechanical concepts were brought up and drawbacks were seen [23-25]. Early investigations about the Acroflex-Disc, for example, revealed the problem of rubber-tears and made an instant product withdrawal necessary. This has caused a situation of uncertainty during a time of a noticeable increase in usage of lumbar TDR. Finally, one widely noticed article raising concerns [26] led the Swiss Federal Office of Public Health to temporarily link the reimbursement of TDR to participation in a Health Technology Assessment (HTA) registry. Following the governmental request for close monitoring of all TDR procedures, a nationwide registry was implemented according to the principle of "coverage with evidence development" [27,28]. At the same time, when spine surgery figures in the USA were increasing exponentially, Deyo et al. called for rigorous post marketing surveillance of adverse events for all new implants and procedures [29,30]. Moreover, the ongoing investigations in the USA concerning the use of SB Charité seem to justify these measures [31].

As a nationwide data collection project the SWISSspine registry opened opportunities for investigations with a potentially high external validity. Amongst the most interesting topics were indications and contraindications for lumbar TDR. In the framework of the registry it was left to the surgeons' discretion to operate on patients with HNP and/or radiculopathy. Taking into account the potentially wide range of cases and symptoms we hypothesized that there are differences in outcomes between patients with respect to the preoperative NP status and the presence or absence of radiculopathy. Furthermore complications and revision rates are displayed because we assumed to find differences in the four groups based on different extents of decompression.

## Methods

Between March 2005 and April 2009, 768 patients with lumbar TDR were documented. The following implants were used: ActiveL (Braun/Aesculap, Tuttlingen, Germany); Dynardi (Zimmer, Warsaw, IN, USA); Maverick (Medtronic Sofamor Danek, Memphis, TN, USA); ProDisc II (Spine Solutions/Synthes, Paoli, PA, USA); SB Charité (DePuy Spine, Raynham, MA, USA). Following predefined criteria for surgery given by the Swiss Spine Society SGS, the TDR was only implanted after an at least 6 months unsuccsessful conservative therapy.

Inclusion criteria for the study were monosegmental surgery and availability of a follow-up examination between 1 and 3 years after surgery. According to the inclusion criteria, 120 bisegmental patients and 188 patients who had not yet reached the 1 year follow-up were excluded. Further, 27 excluded patients did not have any follow-up records yet.

Consequently 433 patients with a mono-segmental intervention were included in the current study. There were 174 (40%) male and 259 (59%) female patients with a mean age of 41 years (SD 9.5 years; range 19 - 65 years) and 43 years (SD 9 years; range 20 - 65 years), respectively. Each patient had signed informed consent and completed NASS and EuroQol-5D questionnaires, which were stored in the MEMdoc database at the University of Bern's Institute for Evaluative Research in Medicine (IEFM) [32-34]. Furthermore, clinical records for each patient were gathered by the physicians. Mean follow-up time using the latest available follow-up assessment between the 1^st ^and 3^rd ^postoperative year was 1 year and 10 months (SD 8 months).

We compared four patient groups regarding the NP status and presence or absence of radiculopathy (Table 1).

**Table 1:** Group allocation.

	Description	HNP	Radiculopathy	N
DDD	classic precondition	no	no	160
HNP-No radiculopathy	HNP only	yes	no	68
Stenosis	recess stenosis	no	yes	73
HNP-Radiculopathy	classic contraindication	yes	yes	132

Radiculopathy was defined as sciatica (leg pain) with sensory and/or motor deficit and was based on the preoperative examination as recorded by the surgeon.

The group "DDD" consisted of 160 (36.9%) patients with neither HNP nor radiculopathy, which is the classic precondition for TDR. There were 68 (15.7%) patients in the group "HNP-No radiculopathy". The group "Stenosis" comprised 73 patients (16.9%) without HNP but with radiculopathy which we attributed to a recess stenosis or reduced clearance of the foramen. Finally, group "HNP-Radiculopathy" consisted of 132 (30.5%) patients with both, HNP and radiculopathy. The latter could be considered as the group with the typical contraindication.

### Patient based assessment

Pain was assessed using two separate visual analog scales (VAS) for back- and leg pain, both located on the NASS form. General quality of life was assessed using EQ-5D [35,36]. In this cost-utility based instrument values range from -0.6 (quality of life worse than death) via 0 (quality of life equals death) to 1 (best possible quality of life).

### Statistical analysis

Descriptive statistics for patient characteristics were calculated for each group. Comparisons between the groups regarding patient characteristics as well as achievement of minimum clinically relevant difference (MCID) were performed using univariate logistic regressions or analysis of variance, where applicable. Pre- and postoperative values, as well as pre- to postoperative differences of the four groups were compared using general linear modeling. Thereby Bonferroni-Holm adjustments were set to account for multiple testing between the groups. As adjustment factors, the following documented and potentially clinically relevant co-variates were employed for all outcomes: gender (female/male), age (continous variable), preoperative pain medication (yes/no), intervertebral level of the intervention (L2/3, L3/4, L4/5, L5/S1), pharmacologically treated depression (yes/no), type of work (sedentary, physical, housewife, retired, unemployed) and working activity level (unable to work, 10-40%, 50-90%, 100%).

All statistical analyses were conducted using SAS 9.2 (SAS Institute Inc, Cary, NC).

The current analysis was conducted in conformity with the Helsinki Declaration. It is based on a governmentally mandated nationwide HTA evaluation registry and therefore ethical approval was not necessary. All patients, did, however, sign three documents explaining the purpose of data collection and asking for informed consent.

## Results

There were no significant differences between the four groups with respect to mean age, gender distribution, types of work and work ability or preoperative medication and distribution of operated levels (Table 2).

**Table 2:** Patient characteristics at baseline.

	DDD	**HNP-No rad**.	Stenosis	**HNP-Rad**.	
	**n = 160 (36.9%)**	**n = 68 (15.7%)**	**n = 73 (16.9%)**	**n = 132 (30.5%)**	**Group comparison (p-value)**

Mean age (yrs.)	41.8	41.1	42.7	41.4	0.83
Age range (yrs.)	20-65	19-65	24-61	20-62	n.a.
Females (%)	66.3	52.9	54.8	58.3	0.15

Occupat. sedentary work (%)	29.8	31.2	26.5	31.4	
Occupat. physical work (%)	48.6	50.8	52.9	55.1	
Occupat. housewife (%)	13.9	16.4	14.7	9.3	0.59
Occupat. retired (%)	3.5	1.6	1.5	1.7	
Occupat. unemployed (%)	4.2	0	4.4	2.5	

Work capacity 100% (%)	71.2	81.8	58.2	69.5	
Work capacity 50-90% (%)	11.4	12.7	27.3	17.1	0.37
Work capacity 10-40% (%)	3.8	0	3.6	1.9	
Unable to work (%)	13.6	5.5	10.9	11.4	

No preop. medication (%)	3.6	4.7	2.2	1.3	
Preop. NSAIDs (%)	66.3	67.1	67.7	70.5	0.39
Preop. opiates (%)	30.1	28.2	30.1	28.2	

L2/3 (%)*	1.3			0.8	
L3/4 (%)	9.7	2.9	5.6	1.5	
L4/5 (%)	47.1	39.7	45.8	48.5	0.10
L5/S1 (%)	41.9	57.4	48.6	49.2	

*3 cases, not part of the analysis; n.a. - not analysed

### VAS back pain

There were no significant differences between the groups in preoperative back pain levels (Table 3). Regarding postoperative back pain, the group "Stenosis" was different from the group "DDD" (p = 0.004) from the group "HNP-No radiculopathy" (p = 0.014) and from the group "HNP-Radiculopathy" (p = 0.031) in that it had a higher mean back pain level. Accordingly, the pain relief in the group "Stenosis" was different from the group "DDD" (p = 0.003), from the group "HNP-No radiculopathy" (p = 0.003), and borderline significantly different from the group "HNP-Radiculopathy" (p = 0.071) (Table 3). After adjustment for covariates, the group "Stenosis" was different from the groups "DDD" (p = 0.001) and "HNP-Radiculopathy" (p = 0.032), whereas the difference to the group "HNP-No radiculopathy" was bordeline not significant (p = 0.064).

**Table 3:** Outcomes.

	DDD	**HNP-No rad**.	Stenosis	**HNP-Rad**.
Preop. back pain	70.2	72.5	67.8	68.1
Postop. back pain	***28.1***	***27.3***	41.8*	***30.3***
Back pain relief: unadjusted	***42.2***	***45.2***	26.0*	37.8
Back pain relief: adjusted	***49.8***	45.9	32.6*	***45.2***

Preop. leg pain	***52.1***	***47.3***	55.0	62.3*
Postop. leg pain	***20.1***	20.2	31.5*	23.9
Leg pain relief: unadjusted	31.7	27.1	23.5*	***38.4***
Leg pain relief: adjusted	***34.7***	33.4	21.8*	***34.0***

Preop. quality of life	0.326	0.371	0.336	0.350
Postop. quality of life	0.753	0.784	0.702	0.754
Improvement of quality of life: unadjusted	0.427	0.413	0.366	0.404
Improvement of quality of life: adjusted	0.317	0.321	0.271	0.299

Unadjusted and adjusted back and leg pain relief and improvement of quality of life are shown in each group. Asteriks marks the group which was significantly different to another group in bold. As adjustment factors, gender, age, preoperative pain medication, intervertebral level of the intervention, pharmacologically treated depression, type of work and working activity level were employed.

A significantly higher proportion of patients in the group "DDD" (84%) reached the MCID in pain relief of 18 points [37] than in the group "Stenosis" (60.6%) (p = 0.002). The differences between the group "HNP-No radiculopathy" (77.4%) and the group "Stenosis" (60.6%) and between the group "HNP-Radiculopathy" (71.7%) and the group "Stenosis" (60.6%) were not significant (p = 0.16 and p = 0.54, respectively).

### VAS leg pain

Differences in the preoperative VAS leg pain result from group allocation (Table 3).

The "HNP-No rad." group revealed unexpectedly high preoperative leg pain levels but this symptom was obviously well adressed with the procedure. In contrast the "Stenosis" group did not benefit as much as the other groups.

Preoperatively the group "DDD" was different from the group "HNP-Radiculopathy" (p = 0.018) which had the highest leg pain levels. Also, the group "HNP-No radiculopathy" was different from the group "HNP-Radiculopathy" (p = 0.003). Regarding postoperative leg pain, the group "DDD" was different from the group "Stenosis" (p = 0.022) which still had the highest mean leg pain levels.

The group "Stenosis" was different from "HNP-Radiculopathy" (p = 0.023) regarding leg pain relief. After adjustment of covariates the group "Stenosis" was different from the group "DDD" (p = 0.026) and from the group "HNP-Radiculopathy" (p = 0.040). In all scenarios the "Stenosis" group had the lowest leg pain relief.

66.7% of patients in the group "DDD" reached the leg pain relief MCID of 18 points [37]. In the group "HNP-No radiculopathy" there were 56.5%, in the group "Stenosis" 60.1% and in the group "HNP-Radiculopathy" 71.7% of patients who reached the MCID. None of the intergroup comparions regarding the MCID in leg pain were significant. If the group "DDD", "HNP-No radiculopathy" and "Stenosis" was compared with the group "HNP-Radiculopathy" the p-values were 1, 0.25 and 0.77, respectively.

### Quality of Life (EQ-5D)

There were no significant differences in pre- or postoperative EQ-5D scores or in unadjusted or adjusted EQ-5D score improvements between the groups (Table 3).

64.4% of patients in group "DDD" reached the MCID of 0.25 EQ-5D points [37,38]. In the group "HNP-No radiculopathy" there were 62.9%, in the group "Stenosis" 52.9% and in the group "HNP-Radiculopathy" 60.8% of patients who reached the MCID. None of the intergroup comparions regarding the MCID of EQ-5D were significant. If the group "DDD", "HNP-No radiculopathy" and "HNP-Radiculopathy" was compared with the group "Stenosis" the p-values were 0.65, 1 and 1, respectively.

### Complication and revision rates

Eight revisions during the same hospital stay were documented; 4 in group "DDD" (2.5%), 1 in group "Stenosis" (1.5%) and 3 in group "HNP-Radiculopathy" (2.3%). Reasons for revisions were the documented intraoperative complications. For one revison in group "DDD" the reason was missing. Details can be seen in Table 4.

**Table 4:** Complications.

	DDD	**HNP-No rad**.	Stenosis	**HNP-Rad**.
Blood vessel injury	3	1	2	6

Ureter injury	-	1	-	-

Vertebral body injury	-	-	1	-

Dura lesion	-	-	-	1

Total (%)	3 (1.8%)	2 (2.9%)	3 (4.1%)	7 (5.3%)

Additionally, when considering the postoperative period until the third follow-up year, there were 6 patients in the group "DDD" (3.8%), 1 in group "HNP-No radiculopathy" (1.5%), 2 in the group "Stenosis" (2.7%), and 9 patients in the group "HNP-Radiculopathy" (6.8%) who were revised during an additional hospitalization. 2 out of 9 revised patients in the group "HNP-Radiculopathy" were revised twice each.

## Discussion

Based on several studies the FDA approved lumbar TDR with some exclusion criteria and many authors considered herniated nucleus pulposus with neural compression as contraindication [39-41]. Others, however, like Zigler et al. [21] described TDR as an option in these patients. In general indications and contraindications have never been consequently adapted to newer results.

With data based on widely used and validated outcome instruments (EQ-5D, NASS-VAS) and on information recorded by surgeons pre- and postoperatively we can draw a relatively clear image of the impact of TDR-surgery on patients' quality of life.

Unfortunately this study, based on data from a case series, cannot provide further information when to perform simple discectomy or when to perform anterior decompression combined with TDR, which has to be regarded as a limitation. Apart from the debate about the superiority of microdiscectomy versus standard limited laminotomy Weber showed good results after 4 years for patients operated on lumbar disc herniation [42]. Latest findings are reported in several studies resulting from the SPORT (Spine Patient Outcome Research Trial) and can confirm these results [43,44].

This study hypothesized that there are outcome differences between the four patient groups defined by HNP status and presence or absence of radiculopathy. We found relatively homogenous extents of back pain relief between three of the four study groups of which only the group DDD had the classic indication for TDR. The group "Stenosis" stands out with clinically relevant and considerably lower back pain relief. Similarly, postoperative leg pain levels were not significantly different between the groups, with the exception of the "Stenosis" group that showed the lowest relative leg pain alleviation. These findings challenge some of the previous limitations for TDR while confirming others.

There were no differences in complication rates. We expected to find a higher number of dura lesions in group HNP-Radiculopathy in which it is essential to go far back past the annulus fibrosus for a full decompression.

Recent publications focus on TDR as a sound 21^st ^century approach. Hopes and appraisals but even more polemics can be found [26,45,46]. Ross et al. evaluated 226 SB Charité III disc implantations in 160 patients concluding that "These poor results indicate that further use of this implant is not justified" [47]. In contrast, Mayer et al. and Siepe et al. described good results for the ProDisc II even applying less invasive approaches [48-50]. Since the alarming results of the Acroflex-Disc study [51] and the ongoing US court cases concerning the SB-Charité [31] the Swiss health care authorities were reluctant to accept TDR as a safe and efficacious therapy. Therefore, a nationwide registry was mandated to closely monitor TDR in uncontrolled clinical settings [28,52]. When SWISSspine was in its role-out phase Mirza commented on important issues like polyethylene debris, loosening, infection, and bone-implant interface [53] and concluded that hopes for a cure of back pain and a marketing bonanza must be held in check by principles of fairness and responsibility and by the long-term results [53].

Published reports like that of Chin et al. [15] suggested that 95% of patients undergoing lumbar spinal surgery had at least one contraindication for TDR. These conclusions do, however, depend upon the definitions of indications and contraindications for lumbar TDR.

Those presented in this study, although based on the current literature, are not universally accepted, like the HNP with neural compression [21].

Indications and contraindications will continue to evolve with the improved understanding of this procedure. Changing them might allow conceivably more patients to be considered for lumbar TDR [18]. Is this desirable? The immediate perceived success obtained with the BAK cage for symptomatic DDD created an environment in which indications were also loosely modified and suboptimal surgical candidates were stabilized with accordingly poorer results. Few surgeons now perform anterior lumbar interbody fusions with stand-alone cages (ALIF), but this procedure is increasingly being abandoned because surgeons have seen it fail frequently [54,55]. This scenario should not be repeated with lumbar TDR.

As lumbar discectomy is a classic procedure for lumbar disc herniation and has been shown to be effective [42], lumbar TDR can currently not compete with the reported longterm results [56]. In the short term perspective, however, the procedures seem to be equal. In accordance to our clinical experience relief of leg pain is a striking clinical factor in microdisectomy [44] as well as in anterior decompression in combination with lumbar TDR consistent to the findings in this study. The indication group most severely drawing our attention to its comparably poor outcomes is the "Stenosis" group. Despite being the second smallest patient group, it still represents about 17% of cases. This group is defined on the absence of HNP and radiculopathy rather than on the presence of other degenerative processes which makes a more precise description of the truly underlying pathology difficult. We interpreted this constellation as a recess and/or foraminal stenosis, and, given the persisting postoperative back and leg pain levels, a degeneration of the posterior bony structures in the sense of a facet joint arthrosis with spondylogenic pain is also very likely. Hence these above listed contraindications are rather confirmed by the "Stenosis" group and lumbar TDR seems a suboptimal solution for these types of patients.

SWISSspine is a prospective observational study of a large cohort of TDR recipients. Using the latest available follow-up record of each patient between the 1^st ^and 3^rd ^followup years we found considerable differences between patients with the typical indication for the procedure and other patients with contraindications like recess stenosis or facet joint arthosis. On the other hand HNP or radiculopathy proved as contraindications with only minor negative influence on back and leg pain relief. Although German and Foley compared the literature concerning both TDR and standalone ALIF trying to identify parallels [57], they concluded that the given obstacles are difficult to overcome.

To date ALIF seems to be the only direct comparator for TDR but it is a weak one, especially regarding biomechanics. The hurdles to overcome when creating and running RCTs comparing TDR to ALIF are high, but it was done by Geisler, Guyer, Blumenthal or McAfee [19-21]. Their results showed a similar patient satisfaction and a slight superiority for TDR in several socio-economic areas such as work status and duration of hospitalization. Studies comparing the various TDR types are upcoming but still rare [58].

Our study has several limitations. The patient classification is based on presence but also on complete absence of HNP and radiculopathy leaving some room for interpretation about the true source of the preoperative symptoms. Besides, follow-up times are moderate compared to other studies [11,48] and the investigation is based on registry data. Therefore limitations have to be considered in the analysis and interpretation of such data. Invalid conclusions can result when insufficient attention is paid to issues such as missing data, sources of bias and data quality. There were no data collected for sub-classifying the HNP and the definition of the symptom 'radiculopathy' is widespread and examiners' subjectivity has influence on that variable; in SWISSspine the surgeon determines if a radiculopathy is present or not. If it was indicated as present but no HNP was seen, we interpreted this as reduced clearance of the foramen causing radiculopathy. On the other hand, a previous analysis revealed a very stable course of postoperative back pain and an even ongoing further improvement of leg pain until 400 days after surgery [28].

Although no common definition of radiculophathy in the literature can be found our own clinical experience tells us that approximately one third of patients presenting with leg pain clearly fulfills criteria for radiculopathy, one third shows mixed signs of radicular and pseudoradicular symptoms, and one third is clearly pseudoradicular. In the literature there is little information on this issue. However, data collection in SWISSspine is multicentric and due to its setting the registry reaches a very high level of representativeness, i.e. external validity and the likelihood of selection bias of patients is low.

The adjustment of mean pain relief and improvement of quality of life in the groups was performed based on the documented variables. Other potentially relevant clinical variables like smoking and duration of symptoms were not part of the already high documentation burden for surgeons, accordingly not allowing for their adjustment. However, only patients with at least 6 months of unsuccessful conservative treatment were operated and documented.

## Conclusions

Despite a HNP and preoperative radiculopathy short term outcomes were similar to patients in the group with the classic indication (DDD). Furthermore these patients did not show inferior outcomes compared with historical cohorts treated with posterior decompression procedures. Patients with recess and/or lateral stenosis, eventually accompanied by facet joint arthrosis showed suboptimal outcomes. Consequently, some of the current agreements on indications and contraindications for lumbar TDA are challenged while others are confirmed and our findings should be validated in trials with higher evidence levels.

## List of abbreviations used

HNP: herniated nucleus pulposus; TDR: total disc replacement; DDD: degenerative disc disease; FDA: the Food and Drug Administration; MRI: Magnetic Resonance Imaging; CT: Computed Tomography; RCTs: randomized controlled trials; n.a.: not analysed; HTA: Health Technology Assessment; MCID: minimum clinically relevant difference.

## Competing interests

The authors declare that they have no competing interests.

## Authors' contributions

TZ is the principal investigator. He initiated and performed the study and drafted the manuscript. CH performed literature review and helped in drafting manuscript. EA performed statistical analysis and helped in drafting the manuscript. MM and CR supervised the study, interpretation of the results and drafting the manuscript. CE supervised interpretation of the results. All authors participated in the study design as well as read and approved the final manuscript.

## Pre-publication history

The pre-publication history for this paper can be accessed here:

http://www.biomedcentral.com/1471-2474/12/275/prepub
